# Novel *Stenotrophomonas maltophilia* temperate phage DLP4 is capable of lysogenic conversion

**DOI:** 10.1186/s12864-019-5674-5

**Published:** 2019-04-16

**Authors:** Danielle L. Peters, Jaclyn G. McCutcheon, Paul Stothard, Jonathan J. Dennis

**Affiliations:** Department of Biological Sciences, 6-065 Centennial Centre for Interdisciplinery Science, University of Alberta, Edmonton, Alberta T6G 2E9 Canada

**Keywords:** *Stenotrophomonas maltophilia*, Antibiotic resistance, Bacteriophage, Temperate phage, Prophage, Phage receptor, Swarming motility

## Abstract

**Background:**

Temperate bacteriophages are capable of lysogenic conversion of new bacterial hosts. This phenomenon is often ascribed to “moron” elements that are acquired horizontally and transcribed independently from the rest of the phage genes. Whereas some bacterial species exhibit relatively little prophage-dependent phenotypic changes, other bacterial species such as *Stenotrophomonas maltophilia* appear to commonly adopt prophage genetic contributions.

**Results:**

The novel *S. maltophilia* bacteriophage DLP4 was isolated from soil using the highly antibiotic-resistant *S. maltophilia* strain D1585. Genome sequence analysis and functionality testing showed that DLP4 is a temperate phage capable of lysogenizing D1585. Two moron genes of interest, *folA* (BIT20_024) and *ybiA* (BIT20_065), were identified and investigated for their putative activities using complementation testing and phenotypic and transcriptomic changes between wild-type D1585 and the D1585::DLP4 lysogen. The gp24 / *folA* gene encodes dihydrofolate reductase (DHFR: FolA), an enzyme responsible for resistance to the antibiotic trimethoprim. I-TASSER analysis of DLP4 FolA predicted structural similarity to *Bacillus anthracis* DHFR and minimum inhibitory concentration experiments demonstrated that lysogenic conversion of D1585 by DLP4 provided the host cell with an increase in trimethoprim resistance. The gp65 / *ybiA* gene encodes N-glycosidase YbiA, which in *E. coli* BW25113 is required for its swarming motility phenotype. Expressing DLP4 *ybiA* in strain *ybiA*770(del)::kan restored its swarming motility activity to wildtype levels. Reverse transcription-PCR confirmed the expression of both of these genes during DLP4 lysogeny.

**Conclusions:**

*S. maltophilia* temperate phage DLP4 contributes to the antibiotic resistance exhibited by its lysogenized host strain. Genomic analyses can greatly assist in the identification of phage moron genes potentially involved in lysogenic conversion. Further research is required to fully understand the specific contributions temperate phage moron genes provide with respect to the antibiotic resistance and virulence of *S. maltophilia* host cells.

## Background

*Stenotrophomonas maltophilia* is an aerobic Gram-negative bacterium ubiquitous in aqueous environments, soils, plants and it is also frequently isolated from hospital settings [1]. Species of the *Stenotrophomonas* genus are very diverse in their phenotypes, genotypes, and ecological niches [2]. Due to this extensive diversity but conserved 16S rRNA gene sequences, these bacteria have been referred to as the *S. maltophilia* complex (SMC) [3]. Although *S. maltophilia* has been useful in biotechnology as a biocontrol of plant pathogens and for bioremediation, an increase in nosocomial and community-acquired *S. maltophilia* infections is causing concern [1]. *S. maltophilia* is capable of causing a variety of infections such as pneumonia, bacteremia, meningitis, endocarditis and catheter-related bacteremia/septicemia [4]. Infection prevention has been difficult as identification of reservoirs and transmission modes has yet to be elucidated [1, 4].

Once infected with *S. maltophilia*, there are few treatment options due to its innate multidrug resistance to a broad array of antibiotics [1]. As an alternative, investigation into the use of bacteriophages to treat *S. maltophilia* infections is currently in the preliminary stages, focusing on phage isolation and characterization [5–17]. When considering the use of phages to treat *S. maltophilia* infections, it is important to note that temperate phages can play a role in horizontal gene transfer of antibiotic resistance and virulence genes between bacteria. Of the 15 *S. maltophilia* phages characterized to date, six encode identifiable moron genes; four phages encode a zot-like protein [13, 15, 16], one phage encodes GspM, a protein involved in the general secretion system [10], and one phage encodes a membrane-modification WecA homologue [17]. Even with a carriage rate of moron genes in characterized *S. maltophilia* phages at 40%, little research has been performed concerning the role of temperate phages in the lysogenic conversion of members of the SMC.

## Methods

### Bacterial strains and growth conditions

Five *S. maltophilia* and eight *P. aeruginosa* strains were obtained from the Canadian *Burkholderia cepacia* complex Research and Referral Repository (Vancouver, BC). The *S. maltophilia* strain used to propagate DLP4 from the soil sample was D1585. An additional 22 *S. maltophilia* strains were obtained from the Provincial Laboratory for Public Health - North (Microbiology), Alberta Health Services, for host range analysis. *Escherichia coli* strains BW25113 and *ybiA*770(del)::kan strains [18] were used for swarming motility experiments and data from three independent biological and mechanical triplicates was used. All strains were grown aerobically overnight at 30 °C on Lysogeny broth (LB or ½ LB) solid media or in LB or ½ LB broth with shaking at 225 RPM. Chloramphenicol at a final concentration of 35 μg/ml was added when required.

### Phage isolation, propagation, host range analysis and electron microscopy

Bacteriophage DLP4 was isolated from a soil sample collected from Emily Murphy Park in Edmonton, Alberta, Canada, using established protocols [19]. Briefly, the soil sample was incubated by shaking at 30 °C in ½ LB broth with modified suspension medium (SM) and an *S. maltophilia* D1585 liquid culture. The lysate was clarified by centrifugation and the supernatant was filter-sterilized using a Millex-HA 0.45 μm syringe driven filter unit (Millipore). The lysate was mixed with strain D1585, plated in soft agar overlays, and incubated overnight at 30 °C. Single plaques were each isolated using a sterile Pasteur pipette and suspended in separate microcentrifuge tubes containing 500 μl SM with 20 μl chloroform and rocked 1 h at room temperature. Purified DLP4 was propagated using soft agar overlays: 100 μl overnight culture and 100 μl phage stock incubated 20 min at room temperature, mixed with 3 ml 0.7% ½ LB top agar, overlaid on a plate of ½ LB solid medium, and incubated at 30 °C overnight. High titer stocks were made by overlaying plates of near-confluent lysis with 3 ml SM and incubating > 1 h at room temperature on a platform rocker. Centrifugation of the supernatant for 5 min at 10,000 x g clarified the lysate which was then filter-sterilized using a Millex-HA 0.45 μm syringe-driven filter unit (Millipore) and stored at 4 °C. Host range analysis was performed using 27 clinical *S. maltophilia* and 19 *P. aeruginosa* strains. Soft-agar overlays containing 100 μl liquid bacterial culture were allowed to solidify for 10 min at room temperature and spotted with 10 μl drops of DLP4 at multiple dilutions and assayed for clearing or plaque formation after overnight incubation at 30 °C. For electron microscopy, phage stocks were prepared as described above with the following modifications: ½ LB agarose plates and ½ LB soft agarose was used for overlays, MilliQ-filtered water was used for phage recovery and passed through a 0.22 μm filter. A carbon-coated copper grid was incubated with lysate for 2 min and stained with 4% uranyl acetate for 30 s. Transmission electron micrographs were captured using a Philips/FEI (Morgagni) transmission electron microscope with charge-coupled device camera at 80 kV (University of Alberta Department of Biological Sciences Advanced Microscopy Facility). The capsid diameter, length, and tail length were calculated using Microsoft Excel based on measurements from 10 individual virions obtained using ImageJ software version 1.50i (NIH, Bethesda, MD).

### Phage plaquing assays

DLP4 plaquing ability was determined by spotting on bacterial soft agar overlays as previously described [19]. Briefly, 100 μL of overnight culture was mixed with 3 mL of 0.7% ½ LB top agar, overlaid onto ½ LB agar with or without antibiotics and allowed to solidify at room temperature for 30 min before spotting 5 μL of phage and incubating for 18 h at 30 °C. DLP4 was standardized to 10^10^ PFU/mL on *S. maltophilia* D1585 and tenfold serially diluted in SM media to 10^3^ PFU/mL. Lysogenized cells of *S. maltophilia* strain D1585 were obtained by isolating and purifying DLP4 infected cells from the centers of large plaques with diffuse edges. PCR amplification using DLP4 specific primers p7F 5′-CTGGGCTTCCTTGTCGTAGATATG-3′ (bps 21,667 to 21,690) and p7R 5′-CTAAGGAGACGGAGATGTACCTGAT-3′ (complemented bps 22,388 to 22,412), which bind within the gene for the DLP4 DNA polymerase, indicated the presence of DLP4 in lysogenized D1585 cells as compared to control D1585 cells. Strains D1585 and 280 Δ*pilA* mutants and complemented strains were utilized [20]. Chloramphenicol was used at a concentration of 35 μg/mL for D1585 and 75 μg/mL for strain 280 for complemented strains. Each experiment was repeated in biological and mechanical triplicate.

### Phage DNA isolation, RFLP analysis, and sequencing

DLP4 genomic DNA was isolated from bacteriophage lysate using the Wizard DNA purification system (Promega Corp.) with a modified protocol [21, 22]. A NanoDrop ND-1000 spectrophotometer (Thermo Scientific) was used to determine the purity and concentration of eluted DNA. Restriction fragment length polymorphism analysis (RFLP) was performed using a panel of 36 FastDigest restriction enzymes (Thermo Scientific) and l μg of phage DNA. Reactions were incubated at 37 °C for 45 min and separated on a 0.8% (wt/vol) agarose gel in 1x TAE (pH 8.0). A DNA genomic library was constructed by The Applied Genomics Core at the University of Alberta using a Nextera XT library prep kit and used for paired-end sequencing on a MiSeq (Illumina) platform using a MiSeq v2 reagent kit.

### Bioinformatic analysis

Paired-end reads were assembled using SPAdes 3.8.0 [23]. Open reading frames (ORFs) were identified using the GLIMMER plugin [24] for Geneious [25] using the Bacteria and Archaea setting as well as the Gene MarkS [26] program for phage. Conserved domain searches were performed using CD-Search [27]. The contig was annotated using BLASTn and BLASTP (for full genomes and individual proteins, respectively) [28]. BLASTX and PHAST [29] were used to search for similar sequences in the GenBank database. Lysis protein analysis was performed using TMHMM for transmembrane region identification [30] and LipoP 1.0 for the prediction of lipoproteins [31]. Protein structure prediction was accomplished using I-TASSER [32]. Protein comparisons were accomplished using MUSCLE [33].

### Swarming motility analysis

Six strains were constructed using two different plasmids (pBBR1MCS [34] and pYbiA) to determine what effect phage-encoded *ybiA* has on swarming (Table 1). Experimental data was obtained from three biological and mechanical triplicate experiments using overnight cultures grown at 30°C in 5 ml lysogeny broth (LB) and supplemented with 35 μg/ml chloramphenicol (Sigma Aldrich). Fresh M8 agar plates [35] were poured and allowed to set for 60 min, then inoculated with 5 μl of overnight culture in the center of the plate. Plates were stacked two high and incubated at 30°C for 24 h followed by room temperature incubation for another 24 h. Plates were photographed at 24 and 48 h without automatic focus to ensure the scale did not change between plates. Images were analyzed using ImageJ software [36] to measure the total area of the swarming bacteria on the plate.

**Table 1:** Bacterial strains and plasmids used in this study

Strain	Description	Source
*Escherichia coli* BW25113	Wildtype control for Keio library	Ref. [18]
*Escherichia coli ybiA*770(del)::kan	ybiA deletion strain	Ref. [18]
*Escherichia coli* DH5α	Sub-clone host	Ref. [36]
*Stenotrophomonas maltophilia* D1585	Wildtype host for DLP4	CBCCRRR^a^
*Stenotrophomonas maltophilia* D1585 Δ*pilA*	Clean deletion of *pilA* in D1585	Ref. [20]
*Stenotrophomonas maltophilia* 280	Wildtype host for DLP4	PLPHN/AHS^b^
*Stenotrophomonas maltophilia* 280 Δ*pilA*	Clean deletion of *pilA* in 280	Ref. [20]
D1585::DLP4	*S. maltophilia* D1585 lysogen with DLP4 prophage	This study
pBBR1-MCS	pBBR1MCS broad-host range cloning vector	Ref. [34]
pD1585*pilA*	pBBR1MCS carrying D1585 *pilA,* Cm^R^	Ref. [20]
p280*pilA*	pBBR1MCS carrying 280 *pilA*, Cm^R^	Ref. [20]
pYbiA	pBBR1MCS carrying DLP4 *ybiA*, Cm^R^	This study
pFolA	pBBR1MCS carrying DLP4 *folA*, Cm^R^	This study

^a^Canadian *Burkholderia cepacia* complex Research and Referral Repository

^b^Provincial Laboratory for Public Health—North, Alberta Health Services

### FolA functionality

Four strains were constructed using *E. coli* DH5α [37], *S. maltophilia* D1585 and two plasmids pBBR1MCS [34] and pFolA to study the functionality of the DLP4 encoded *folA* (Table 1). pFolA was constructed by amplifying the dihydrofolate reductase gene from DLP4 using PCR primers FolA XbaI-F (5′-ATATATTCTAGAGAGCTCGAAGTACAGTCCATTCC-3′) and FolA HindIII-R (5′-ATATATAAGCTTGCATACCTATCACCTACATTGTGGA-3′), and cloning the resulting ~ 680 bp fragment into pBBR1MCS [34] similarly digested with XbaI and HindIII. The resulting plasmid pFolA was cloned into *E. coli* and its DNA sequence was confirmed as correct. The pFolA plasmid properly orients the *dhfr* gene behind the *lacZα* promoter, which provides moderate expression in the media used. Triplicate minimal inhibitory concentration (MIC) experiments were performed using established protocols [38]. Overnight cultures were grown at 30°C in 5 ml lysogeny broth (LB) with 35 μg/ml chloramphenicol. A 1:100 subculture was grown at 30°C to an OD600 of 0.1 in Mueller-Hinton broth (MH) (approximately 2.5 h) and used in 96 well plates containing a trimethoprim dilution series (MP Biomedicals). Following a 16-h incubation, OD600 was observed using a Wallac 1420 VICTOR2 multilabel counter (PerkinElmer, Waltham, MA). Statistical analysis was conducted using GraphPad Prism 7 (GraphPad Software Inc., San Diego, CA) to perform a two-way analysis of variance (ANOVA) with Sidak’s multiple comparison and *P*-values < 0.05 were documented.

### RNA isolation and cDNA synthesis

A modified Epicentre Technologies: MasterPure™ RNA purification protocol [39] was used to isolate total RNA from *S. maltophilia*. Triplicate 5 ml cultures of *S. maltophilia* D1585 and lysogen D1585::DLP4 were grown in LB at 30 °C overnight and used for a 1:100 subculture into 10 ml LB at 30 °C for 4 h (~ 3.0 × 10^8^ CFU/ml). At the point of harvest, a 1.25 ml aliquot of ice-cold ethanol/phenol stop solution (5% water-saturated phenol, pH < 7) was added to the 10 ml culture. Cells were then pelleted by centrifugation at 5000 x g for 10 min and resuspended in 75 μl LB. A 25 μl aliquot of the suspension was transferred into three nuclease-free microcentrifuge tubes. A master mix was made using 3.5 μl Proteinase K (50 μg/μl) into 1 ml of Tissue and Cell Lysis Solution (Epicentre Technologies). A 300 μl aliquot of the master mix was added to each of the three nuclease-free tubes containing the resuspended bacterial culture and thoroughly mixed. The samples were incubated at 65 °C for 15 min with vortexing every 5 min. Following the 65 °C incubation, the cells were iced for 5 min, then 175 μl of MPC Protein Precipitation Reagent (Epicentre Technologies) was added to each tube and vigorously vortexed for 10 s. Particulates were pelleted by centrifugation for 10 min at > 10,000 x g. An additional 25 μl of the MPC solution was added to the tubes which had a clear, small or loose pellet. Following centrifugation, the supernatant was transferred to a new nuclease-free tube with 500 μl isopropanol and inverted 30–40 times. The RNA was pelleted at 4 °C for 10 min at max rpm, followed by removal of the isopropanol layer. The pellet was then rinsed with 1 ml of 75% EtOH, centrifuged briefly and resulting EtOH/isopropanol was removed with a pipette. One RNA pellet was resuspended in 100 μl nuclease-free water then transferred to the second and third tube to resuspend all three pellets in the 100 μl water. A 10 μl aliquot of 10x DNase I buffer (Ambion) and 10 units of RNase-free DNase I was added to the resuspended RNA solution and incubated at 37 °C for 10 min. The reaction was stopped with 5 μl of 50 mM EDTA, and 1 μl of SUPERase-IN (Ambion) was added. The resulting purified RNA was quantified then aliquoted into single-use tubes for storage at − 80 °C.

cDNA was synthesized using a modified protocol from GeneChip™ Expression Analysis with specific protocols for the GeneChip™ [40]. The RNA concentrations were standardized to 500 ng/μl, and 3.5 μg of total RNA was used for the reactions. A 4 μl aliquot of random hexamers (Invitrogen) and 1 μl of dNTPs (10 mM) was dispensed into the tubes containing RNA, and the final volume was adjusted to 12 μl with RNase-free water. This mix was incubated at 70 °C for 10 min, followed by 25 °C for 10 min and then chilled to 4 °C and briefly centrifuged. To this reaction mixture, 4 μl of 5x first strand buffer, 2 μl 0.1 M DTT, 1 μl SUPERase-IN and 1 μl SuperScript II (SSII). The solution was gently mixed and centrifuged, followed by these incubation steps: 25 °C 10 min, 37 °C 60 min, 42 °C for 60 min, and inactivation of SSII at 70 °C for 10 min, then chilled to 4 °C. The resulting mixture was cleaned up with a QIAquick PCR Purification Kit (Qiagen) with a 40 μl elution.

### Reverse transcription PCR

PCR analysis was conducted on each purified cDNA isolate using TopTaq DNA polymerase, buffer, and Q-solution (Qiagen), as well as primers specific to each gene of interest (Integrated DNA Technologies). Positive control primers were designed for *S. maltophilia* D1585 r*poD* (RNA polymerase sigma factor RpoD) and *proC* (proline biosynthetic gene). Gene-specific primers were designed from the DLP4 genome to detect *folA* (dihydrofolate reductase), *ybiA* (N-glycosidase), and *cas4*. The amount of cDNA used in each reaction was standardized to 200 ng. The resulting products were separated on a 1% (wt/vol) agarose gel in 1x TAE (pH 8.0) and stained with ethidium bromide for visualization with a ChemiDoc MP imaging system and the Image Lab software (Bio-Rad).

## Results and discussion

### Isolation, host range, and morphology

**Fig. 1:**
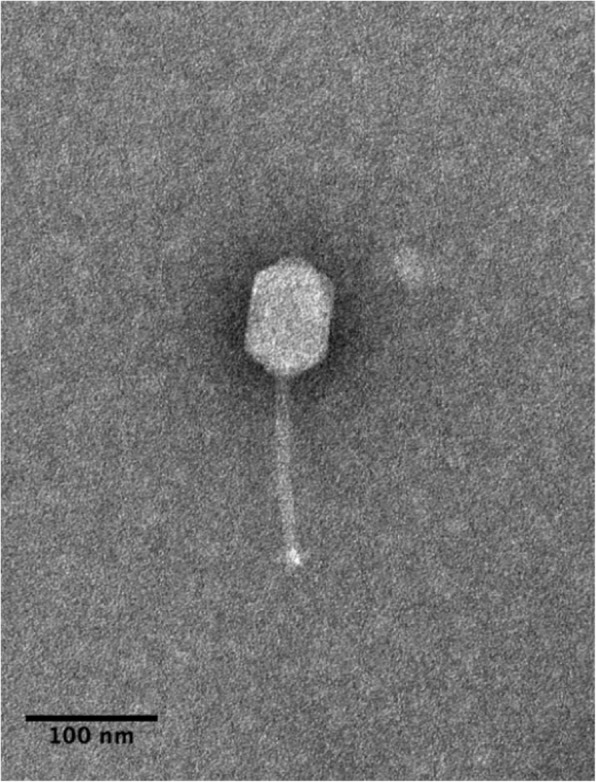
*Siphoviridae* phage DLP4. DLP4 lysate was stained with 4% uranyl acetate on a copper grid and viewed with a Philips/FEI transmission electron microscope. Scale bar represents 100 nm. Measurements of 10 DLP4 phages provide capsid width and length averages of 63 and 92 nm respectively, and a tail length average of 139 nm

Bacteriophage DLP4 (vB_SmaS-DLP_4) was isolated from asparagus soil in Edmonton, Alberta, Canada using clinical *Stenotrophomonas maltophilia* strain D1585. Electron microscopy of DLP4 (Fig. 1) shows that it has a long, noncontractile tail averaging 139 nm and capsid width and length of 63 and 92 nm respectively. The capsid width to length ratio is 1.46, classifying it as a B2 morphotype [41] of the *Siphoviridae* family and the *Caudovirales* order. Host range analysis showed DLP4 is capable of lytic growth on 14 of 27 clinical *S. maltophilia* isolates (Table 2). Although DLP4 is most closely related to *Pseudomonas aeruginosa* phages AAT-1, PaMx28 and PaMx74 at the nucleotide level, host range analysis of DLP4 on *P. aeruginosa* strains showed that it is not capable of infecting the *P. aeruginosa* strains tested [42]. Plaque development by DLP4 occured readily at 30 °C within 16 h, forming diffuse plaques with irregular borders and a mean size of 0.8 ± 0.3 mm.

**Table 2:** Host range analysis of phage DLP4 against *Stenotrophomonas maltophilia* and *Pseudomonas aeruginosa* strains and isolates

	Efficiency of plating
*S. maltophilia* strains	
101^c^	++
102^c^	++
103^c^	+++
152^c^	–
155^c^	++++
174^c^	–
176^c^	–
213^c^	–
214^c^	–
217^c^	–
218^c^	–
219^c^	++
230^c^	+
236^c^	–
242^c^	–
249^c^	–
278^c^	+
280^c^	++
282^c^	++++
287^c^	+
446^c^	–
667^c^	+
D1585^a, b^	+++
D1571^a, b^	–
D1614^a, b^	–
D1576^a, b^	++++
D1568^a, b^	+++
*P. aeruginosa* strains	
PA01	–
HER1004	–
HER1012	–
14715	–
Utah3	–
Utah4	–
14655	–
6106	–
pSHU-OTE	–
D1606D^a, b^	–
D1615C^a, b^	–
D1619M^a, b^	–
D1620E^a, b^	–
D1623C^a, b^	–
ENV003^a^	–
ENV009^a^	–
FC0507^a^	–
R285	–
14672	–

–, No sensitivity to phage; +, plaques at 10^− 2^; ++, clearing at 10^− 2^; +++, plaques at 10^− 4^; ++++, plaques at 10^− 6^

^a^Obtained from the Canadian *Burkholderia cepacia* complex Research Referral Repository

^b^Cystic fibrosis patient isolate

^c^Isolates from the Provincial Laboratory for Public Health - North (Microbiology), Alberta Health Services

### Receptor identification

**Fig. 2:**
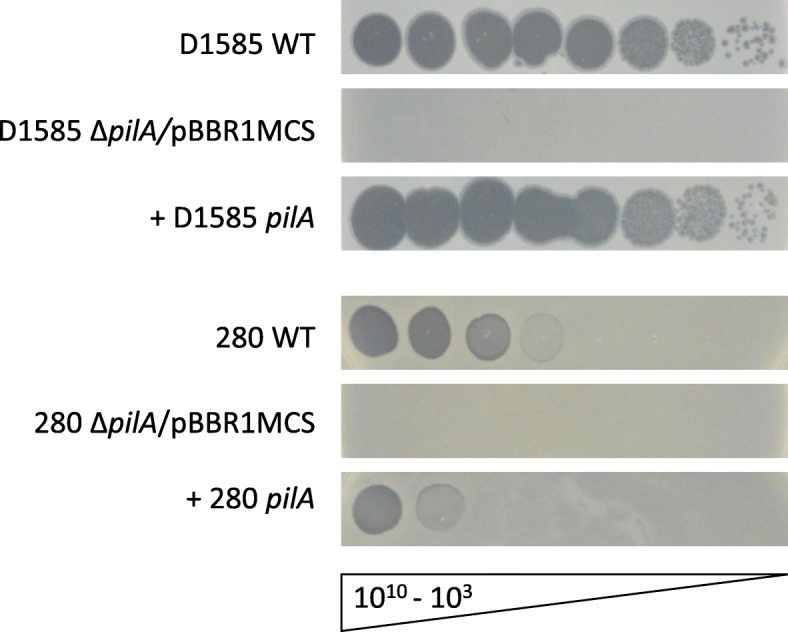
Infection of *S. maltophilia* strains D1585 and 280 by phage DLP4. Wildtype (WT) *S. maltophilia* strains D1585 and 280 are susceptible to DLP4 infection, whereas the Δ*pilA* mutants in both strains are resistant to phage infection. Complementation of strain D1585 Δ*pilA* with the D1585 *pilA* gene restores phage infection to wildtype levels, plaquing at 10^3^ PFU/mL. Complementation of strain 280 Δ*pilA* with the endogenous 280 *pilA* gene partially restores DLP4 infectivity, clearing at 10^9^ PFU/mL compared to 10^7^ PFU/mL on 280 WT. Images are representative of three biological replicates, each with three technical replicates

Two additional *Siphoviridae* bacteriophages, DLP1 and DLP2, previously isolated on *S. maltophilia* strain D1585 and characterized [8] were found to use the type IV pilus as the cell surface receptor for infection across their host range [20]. Assessment of phage DLP4 plaquing ability by spot assay on the previously constructed *S. maltophilia* strains D1585 and 280 Δ*pilA* mutants lacking the major pilin subunit [20] showed similar results; without a functional type IV pilus the mutants are also resistant to DLP4 infection, showing an absence of cell lysis at high titer (Fig. 2). Subsequent complementation of the D1585 Δ*pilA* mutant with the endogenous *pilA* gene restores infection to wildtype levels, producing plaques at 10^3^ PFU/mL. DLP4 has a lower efficiency of plating on strain 280, clearing at 10^7^ PFU/mL, and complementation of the Δ*pilA* mutant with the endogenous gene partially restores infectivity, showing clearing at 10^9^ PFU/mL (Fig. 2). Transformation of each mutant with an empty pBBR1MCS vector did not restore phage infection and no change in bacterial growth in each phage spot was observed. These results identify the type IV pilus as essential for DLP4 infection of *S. maltophilia* strains.

The type IV pilus is a virulence factor on the surface of many bacteria, making it a common receptor target for many bacteriophages. DLP4 is the third *S. maltophilia Siphoviridae* bacteriophage identified to require the type IV pilus for productive cell infection.

### Genome characterization

**Fig. 3:**
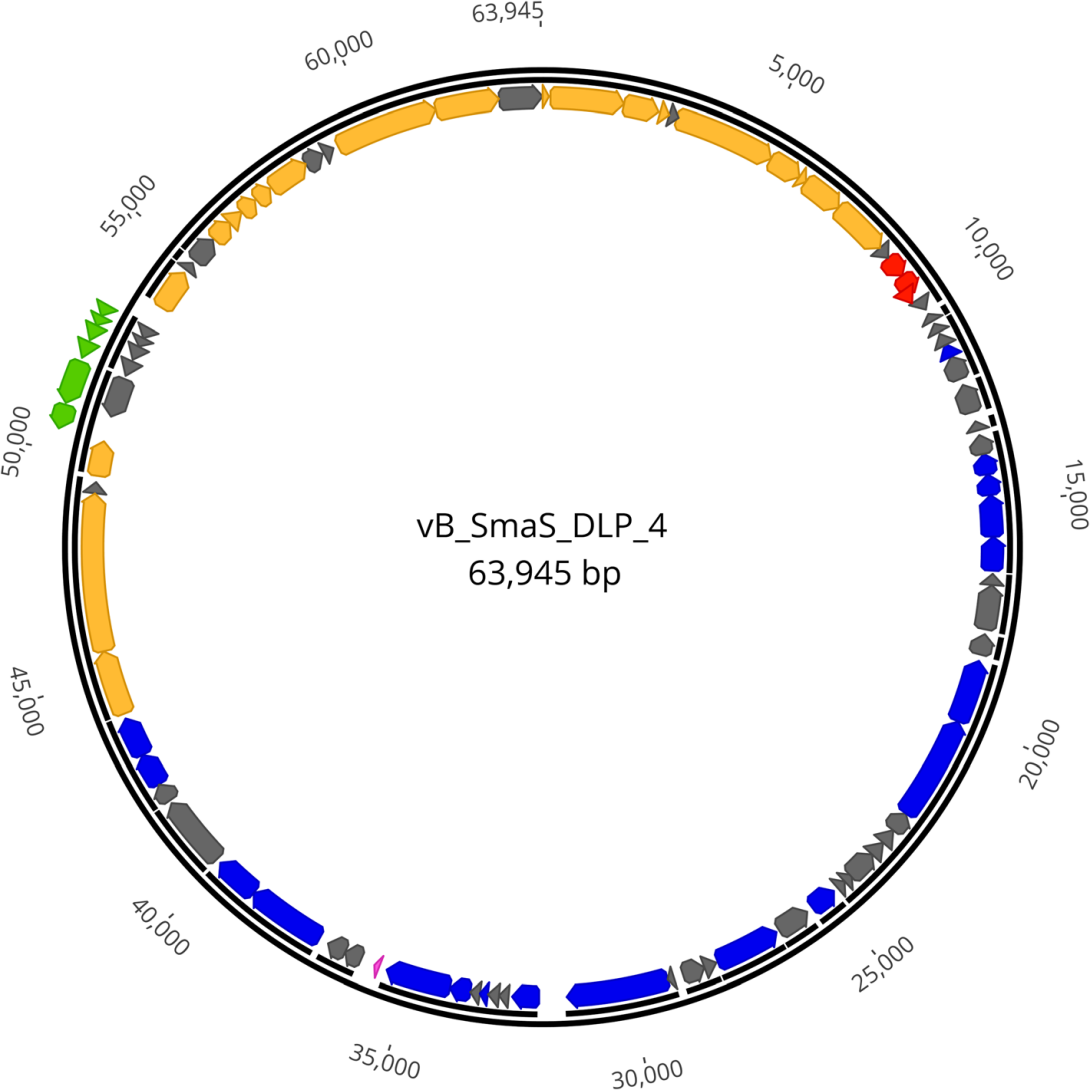
Circularized genome map of DLP4. The scale in bp is shown on outer periphery. Assigned functions for each predicted open reading frame are as follows: lysis; red, virion morphogenesis; mustard, DNA replication/repair; blue, hypothetical; grey, and YbiA operon; light green

Restriction fragment length polymorphism (RFLP) analysis on purified gDNA was unsuccessful because 36 restriction enzymes tested failed to digest the genomic DNA. Restriction enzyme resistant DNA was also found with *P. aeruginosa* phages PaMx28 and PaMx74 [43]. Although the panel of restriction enzymes was smaller (Ndel, HindIII, and EcoRI), the authors did notice a general trend of restriction enzyme resistant DNA in the other 17 phage B2 morphotypes studied [43]. DLP4 assembled into a 63,945 bp circular contig with a read coverage of 1928 and a 100% Q40. The contig was confirmed with PCR using 15 primer sets followed by Sanger sequencing. The DLP4 genome has a GC content of 65% and is predicted to encode 82 ORFs (Fig. 3, Table 3). The modular arrangement of genes based on function shows distinct regions encoding genes involved in DNA replication and repair (dark green), lysis (red), virion morphogenesis (blue) and the YbiA operon (green) (Fig. 3). Although DLP4 was confirmed to be a temperate phage capable of establishing a lysogenic infection within *S. maltophilia* D1585, the repressor and integrase could not be identified using BLASTP, CD-Search or Pfam. The genome sequence of DLP4 deposited in GenBank has the accession number MG018224.

**Table 3:** Genome annotation of bacteriophage DLP4

Gene	Coding region	Length (AA)	Strand	Start codon	Putative function	BLASTp hit	Query coverage	E value	Identity	Accession
1	5–1135	377	+	ATG	hypothetical protein	virion structural protein [Pseudomonas phage AAT-1]	94%	3.00E-13	62%	AME18051.1
2	1117–2799	561	+	ATG	serine--glyoxylate aminotransferase	putative virion structural protein [Pseudomonas phage PaMx74]	95%	0	63%	YP_009199471.1
3	2799–3611	271	+	ATG	FAD/FMN dehydrogenase	putative virion structural protein [Pseudomonas phage PaMx74]	100%	7.00E-151	73%	YP_009199472.1
4	3624–3857	78	+	ATG	virion structural protein	putative virion structural protein [Pseudomonas phage PaMx74]	98%	1.00E-39	83%	YP_009199473.1
5	3854–4057	68	+	GTG	hypothetical protein	hypothetical protein PaMx74_35 [Pseudomonas phage PaMx74]	98%	7.00E-23	64%	YP_009199474.1
6	4044–6359	772	+	ATG	virion structural protein	putative virion structural protein [Pseudomonas phage PaMx74]	97%	0	58%	YP_009199475.1
7	6359–7138	260	+	ATG	tail assembly protein	tail assembly protein [Xylella phage Salvo]	100%	2.00E-82	51%	AHB12242.1
8	7142–7306	55	+	ATG	tail assembly protein	tail assembly protein [Xylella phage Sano]	98%	1.00E-04	37%	AHB12066.1
9	7316–8260	315	+	ATG	tail assembly protein	tail assembly protein [Xylella phage Salvo]	99%	1.00E-90	51%	AHB12240.1
10	8263–9546	428	+	GTG	hypothetical protein	tail fiber protein [Xylella phage Salvo]	24%	7.00E-12	41%	AHB12239.1
11	9543–9848	102	+	GTG	hypothetical protein	hypothetical protein AAT1_02032 [Pseudomonas phage AAT-1]	98%	7.00E-39	64%	AME18058.1
12	9845–10,339	165	+	ATG	endolysin	putative endolysin [Pseudomonas phage PaMx74]	99%	3.00E-92	83%	YP_009199477.1
13	10,350–10,826	159	+	ATG	i-spanin	virion structural protein [Pseudomonas phage PaMx28]	100%	8.00E-59	63%	YP_009210650.1
14	10,639–11,013	125	+	GTG	o-spanin	putative o-spanin [Pseudomonas phage AAT-1]	96%	2.00E-51	68%	ANN44564.1
15	11,010–11,285	92	+	GTG	hypothetical membrane protein	putative membrane protein [Pseudomonas phage PaMx74]	98%	2.00E-37	67%	YP_009199480.1
16	11,417–11,593	59	–	ATG	hypothetical protein	hypothetical protein PaMx74_42 [Pseudomonas phage PaMx74]	98%	4.00E-04	40%	YP_009199481.1
17	11,661–11,891	77	–	ATG	hypothetical protein	hypothetical protein PaMx74_43 [Pseudomonas phage PaMx74]	67%	3.00E-14	63%	YP_009199482.1
18	11,918–12,202	95	–	ATG	hypothetical protein	hypothetical protein AAT1_02038 [Pseudomonas phage AAT-1]	90%	4.00E-04	37%	AME18064.1
19	12,186–12,491	102	–	ATG	HIRAN domain protein	putative HIRAN domain-containing protein [Pseudomonas phage PaMx28]	92%	2.00E-27	57%	YP_009210657.1
20	12,491–13,048	186	–	ATG	hypothetical protein	hypothetical protein PaMx74_46 [Pseudomonas phage PaMx74]	98%	5.00E-58	60%	YP_009199485.1
21	13,171–13,839	223	–	ATG	hypothetical protein	hypothetical protein PaMx74_47 [Pseudomonas phage PaMx74]	98%	7.00E-62	50%	YP_009199486.1
22	14,040–14,264	75	–	ATG	hypothetical protein	hypothetical protein PaMx74_48 [Pseudomonas phage PaMx74]	85%	7.00E-25	76%	YP_009199487.1
23	14,386–14,832	149	–	GTG	hypothetical protein	hypothetical protein PaMx74_49 [Pseudomonas phage PaMx74]	94%	3.00E-70	75%	YP_009199488.1
24	14,817–15,305	163	–	ATG	FolA/DHFR	putative dihydrofolate reductase [Pseudomonas phage PaMx74]	100%	3.00E-59	61%	YP_009199489.1
25	15,290–15,766	159	–	ATG	deoxycytidylate deaminase	putative dCMP deaminase [Pseudomonas phage PaMx74]	92%	6.00E-70	70%	YP_009199490.1
26	15,766–16,707	314	–	GTG	thymidylate synthase	thymidylate synthase [Pseudomonas phage AAT-1]	98%	1.00E-161	71%	AME18072.1
27	16,704–17,477	258	–	ATG	nucleotide pyrophosphohydrolase	putative nucleotide pyrophosphohydrolase [Pseudomonas phage AAT-1]	89%	3.00E-106	63%	AME18073.1
28	17,553–17,783	77	–	ATG	hypothetical protein					
29	17,786–18,805	340	–	ATG	hypothetical protein	hypothetical protein PaMx28_55 [Pseudomonas phage PaMx28]	97%	0	74%	YP_009210667.1
30	18,920–19,384	155	–	ATG	hypothetical protein	hypothetical protein PaMx28_56 [Pseudomonas phage PaMx28]	61%	2.00E-42	78%	YP_009210668.1
31	19,526–21,004	493	–	GTG	helicase	putative helicase [Pseudomonas phage PaMx74]	99%	0	80%	YP_009199496.1
32	21,001–23,376	792	–	ATG	DNA polymerase	putative DNA polymerase [Pseudomonas phage PaMx74]	90%	0	77%	YP_009199498.1
33	23,405–23,851	149	–	GTG	hypothetical protein	hypothetical protein AAT1_02054 [Pseudomonas phage AAT-1]	100%	6.00E-60	57%	AME18080.1
34	23,848–24,237	130	–	TTG	hypothetical protein	hypothetical protein PaMx74_61 [Pseudomonas phage PaMx74]	99%	3.00E-60	75%	YP_009199500.1
35	24,234–24,578	115	–	ATG	hypothetical protein	hypothetical protein PaMx74_63 [Pseudomonas phage PaMx74]	93%	2.00E-54	75%	YP_009199502.1
36	24,575–25,258	228	–	ATG	hypothetical protein	hypothetical protein PaMx74_65 [Pseudomonas phage PaMx74]	99%	1.00E-89	56%	YP_009199504.1
37	25,248–25,448	67	–	GTG	hypothetical protein	hypothetical protein PaMx74_66 [Pseudomonas phage PaMx74]	93%	8.00E-19	58%	YP_009199505.1
38	25,445–25,672	76	–	ATG	hypothetical protein					
39	25,793–26,404	204	–	ATG	DNA binding protein	putative DNA binding protein [Pseudomonas phage PaMx28]	99%	2.00E-108	78%	YP_009210678.1
40	26,538–27,317	260	–	ATG	hypothetical protein	hypothetical protein PaMx28_69 [Pseudomonas phage PaMx28]	77%	1.00E-76	59%	YP_009210681.1
41	27,390–28,868	493	–	ATG	Cas4 nuclease	PD-(D/E) XK nuclease superfamily protein	99%	0	72%	YP_009210682.1
42	28,918–29,181	88	–	ATG	hypothetical protein	hypothetical protein PaMx74_72 [Pseudomonas phage PaMx74]	95%	5.00E-43	81%	YP_009199511.1
43	29,178–29,702	175	–	ATG	hypothetical protein	hypothetical protein PaMx28_72 [Pseudomonas phage PaMx28]	98%	6.00E-23	43%	YP_009210684.1
44	29,904–30,074	57	+	ATG	hypothetical protein	hypothetical protein PaMx74_74 [Pseudomonas phage PaMx74]	89%	6.00E-16	72%	YP_009199513.1
45	30,071–32,392	774	+	GTG	primase	putative primase/polymerase [Pseudomonas phage PaMx74]	99%	0	85%	YP_009199514.1
46	33,021–33,638	206	+	GTG	small terminase	terminase large subunit [Pseudomonas phage AAT-1]	95%	9.00E-108	77%	AME18098.1
47	33,701–33,919	73	+	ATG	hypothetical protein					
48	33,929–34,171	81	+	ATG	hypothetical protein	hypothetical protein PaMx74_02 [Pseudomonas phage PaMx74]	93%	1.00E-27	67%	YP_009199441.1
49	34,168–34,362	65	+	ATG	deoxynucleoside monophosphate kinase	hypothetical protein [Lysobacter sp. Root667]	78%	1.00E-08	56%	WP_056102216.1
50	34,359–34,577	73	+	ATG	hypothetical protein	hypothetical protein [Lysobacter capsici]	76%	2.00E-06	47%	WP_057921118.1
51	34,581–35,045	155	+	GTG	RNA Pseudouradine synthase					
52	35,045–36,514	490	+	ATG	large terminase	putative terminase large subunit [Pseudomonas phage PaMx74]	100%	0	80%	YP_009199443.1
53	37,156–37,560	135	+	ATG	hypothetical protein	hypothetical protein PaMx74_06 [Pseudomonas phage PaMx74]	98%	6.00E-07	34%	YP_009199445.1
54	37,557–37,991	145	+	ATG	hypothetical protein					1
55	38,200–40,020	607	+	ATG	nrdA	nrdA [uncultured Mediterranean phage uvMED]	97%	0	51%	BAQ94146.1
56	40,028–41,020	547	+	ATG	nrdB	nrdB [uncultured Mediterranean phage uvMED]	94%	5.00E-126	54%	BAR25383.1
57	41,131–42,771	331	+	ATG	hypothetical protein	hypothetical protein PaMx74_08 [Pseudomonas phage PaMx74]	99%	4.00E-169	51%	YP_009199447.1
58	42,840–43,274	145	+	ATG	hypothetical protein	hypothetical protein PaMx74_10 [Pseudomonas phage PaMx74]	81%	1.00E-06	40%	YP_009199449.1
59	43,271–44,059	263	+	GTG	polynucleotide kinase	hypothetical protein [Mitsuaria chitosanitabida]	58%	3.00E-38	46%	WP_067070380.1
60	44,059–44,955	299	+	ATG	DNA ligase	putative DNA ligase [Pseudomonas phage PaMx74]	97%	2.00E-106	57%	YP_009199450.1
61	45,042–46,562	507	+	ATG	portal protein	structural protein [Pseudomonas phage AAT-1]	94%	0	78%	AME18030.1
62	46,562–50,131	1190	+	ATG	minor head protein	morphogenesis protein [Pseudomonas phage PaMx28]	100%	0	75%	YP_009210622.1
63	50,133–50,405	91	+	ATG	hypothetical protein	hypothetical protein PaMx28_11 [Pseudomonas phage PaMx28]	95%	1.00E-42	80%	YP_009210623.1
64	50,549–51,334	262	+	ATG	virion structural protein	virion structural protein [Pseudomonas phage AAT-1]	98%	8.00E-146	78%	AME18033.1
65	51,384–51,869	162	–	GTG	YbiA	putative YbiA-like protein [Pseudomonas phage PaMx28]	98%	2.00E-81	72%	YP_009210625.1
66	51,909–52,817	303	–	ATG	hypothetical protein	hypothetical protein PaMx74_15 [Pseudomonas phage PaMx74]	99%	3.00E-70	47%	YP_009199454.1
67	52,903–53,280	126	–	ATG	hypothetical protein	hypothetical protein AAT1_02012 [Pseudomonas phage AAT-1]	73%	2.00E-19	49%	AME18037.1
68	53,292–53,621	110	–	ATG	hypothetical protein	hypothetical protein [Enterobacteriaceae]	65%	0.034	35%	WP_044347588.1
69	53,636–53,848	71	–	ATG	hypothetical protein	hypothetical protein AAT1_02013 [Pseudomonas phage AAT-1]	80%	9.00E-20	71%	AME18038.1
70	53,856–54,095	80	–	ATG	hypothetical protein					
71	54,657–55,589	311	+	ATG	major head protein	major head protein [Pseudomonas phage PaMx28]	99%	0	87%	YP_009210631.1
72	55,657–55,890	78	+	ATG	hypothetical protein					
73	55,957–56,568	204	+	ATG	hypothetical protein	hypothetical protein PaMx74_20 [Pseudomonas phage PaMx74]	99%	5.00E-47	49%	YP_009199459.1
74	56,591–57,115	175	+	ATG	virion structural protein	putative virion structural protein [Pseudomonas phage PaMx74]	99%	1.00E-84	72%	YP_009199461.1
75	57,117–57,485	123	+	ATG	virion structural protein	putative virion structural protein [Pseudomonas phage PaMx74]	100%	2.00E-46	61%	YP_009199462.1
76	57,487–57,879	131	+	GTG	virion structural protein	virion structural protein [Pseudomonas phage PaMx28]	98%	5.00E-72	80%	YP_009210635.1
77	57,892–58,314	141	+	ATG	tail terminator protein	putative tail terminator protein [Pseudomonas phage PaMx74]	97%	6.00E-92	93%	YP_009199464.1
78	58,337–59,278	314	+	ATG	major tail structural protein	major tail structural protein [Pseudomonas phage AAT-1]	100%	0	80%	AME18046.1
79	59,283–59,729	149	+	ATG	hypothetical protein	hypothetical protein AAT1_02022 [Pseudomonas phage AAT-1]	89%	3.00E-54	61%	AME18047.1
80	59,768–60,013	82	+	ATG	hypothetical protein	hypothetical protein PaMx74_28 [Pseudomonas phage PaMx74]	100%	3.00E-46	90%	YP_009199467.1
81	60,117–62,474	786	+	ATG	tape measure protein	putative tail tape measure protein [Pseudomonas phage PaMx74]	99%	0	74%	YP_009199468.1
82	62,490–63,944	485	+	ATG	tail fiber structural protein	tail fiber structural protein [Pseudomonas phage MP1412]	100%	3.00E-98	39%	YP_006561077.1

### DNA replication and repair module

Phage DLP4 encodes 45 genes involved in DNA replication, repair and the generation and processing of nucleotides (BIT20_016–060). Within the module, gene products that could be assigned a function include helicase (BIT20_031), DNA polymerase (BIT20_032), DNA binding protein (BIT20_039), Cas4 nuclease (BIT20_041), primase (BIT20_045), small terminase (BIT20_046), deoxynucleoside monophosphate kinase (BIT20_049), RNA pseudouridine synthase (BIT20_051), large terminase (BIT20_052), NrdA (BIT20_055), NrdB (BIT20_056), polynucleotide kinase (BIT20_059), DNA ligase (BIT20_060) and a protein with a conserved HIRAN domain (BIT20_019)(Fig. 3, Table 3). Proteins with HIRAN domains have been shown to identify DNA damage and stalled replication forks [44], though the functionality of the DLP4 protein is currently unknown.

Identification of a Cas4 nuclease conserved domain within a DLP4-encoded protein warranted further investigation. Phage-encoded Cas4 nuclease homologs were previously identified in *Campylobacter jejuni* bacteriophages and were shown to be capable of inserting new spacers into the CRISPR array of their host bacterium [45]. The spacers incorporated into the array were host-derived, suggesting that the phages make decoy spacers using the Cas4 nuclease to prevent the degradation of phage DNA [45]. BLASTP analysis of the Cas4 nuclease (BIT20_041) reveals this protein is also highly conserved within bacteriophages that infect a range of hosts such as *Acinetobacter, Xanthomonas, Pseudomonas,* and *Achromobacter*. To determine if DLP4 is using the putative Cas4 nuclease to incorporate new host-specific spacers like the *C. jejuni* phages, expression of *cas4* was examined using RT-PCR. However, no expression of *cas4* was observed during the lysogenic phase (data not shown), and attempts to identify CRISPR arrays within a *S. maltophilia* D1585 scaffold using CRISPRCasFinder (https://crisprcas.i2bc.paris-saclay.fr/CrisprCasFinder/Index) were unsuccessful.

A cluster of genes encoded within the DNA module are involved in the generation and processing of deoxyribonucleosides for their immediate use in phage DNA synthesis during the lytic cycle. DLP4 contains the genes *nrdA* and *nrdB* (BIT20_055/056 respectively) that encode the α2 and β2 subunits of ribonucleoside diphosphate reductase (RDR). The RDR protein reduces ribonucleosides to deoxyribonucleosides, the first step in the generation of deoxyribonucleoside triphosphates [46]. The next processing step of the resulting deoxyribonucleoside monophosphates (dNMP) is the addition of a phosphate group to make deoxyribonucleoside diphosphates (dNDP) using either ATP or dATP as the phosphate donor [47]. This step is carried out by the substrate-specific enzyme deoxyribonucleoside monophosphate kinase (encoded by DLP4 BIT20_049), which is substrate specific to dGMP, dTMP, and 5-hydroxymethyl-dCMP [48].

Before all of the dCMP is phosphorylated to its dCDP form, some can be processed by the enzyme dCMP deaminase (deoxycytidylate deaminase: BIT20_025) to produce deoxyuridine monophosphate (dUMP). The dUMP product is the nucleotide substrate for thymidylate synthase (BIT20_026), which produces deoxythymidine monophosphate (dTMP). The thymidylate synthase reaction drives the concomitant conversion of 5,10-methylenetetrahydrofolate to dihydrofolate [49]. The dihydrofolate can then be reduced by DLP4’s dihydrofolate reductase (BIT20_024) into tetrahyrofolic acid, which is processed by serine transhydroxymethylase to regenerate the 5,10-methylenetetrahydrofolate. This enzyme was not identified in DLP4, but the enzyme serine:glyoxylate aminotransferase (BIT20_002) is encoded which catalyzes the reversible reaction of glycine and hydroxypyruvate to produce glyoxylate and L-serine (KEGG reaction: R00588). This L-serine could then be used by the hosts’ serine transhydroxymethylase (encoded by *glyA* in *S. maltophilia*) to regenerate the 5,10-methylenetetrahydrofolate for the dUMP to dTMP reaction catalyzed by thymidylate synthase.

### Investigation of dihydrofolate reductase functionality

**Fig. 4:**
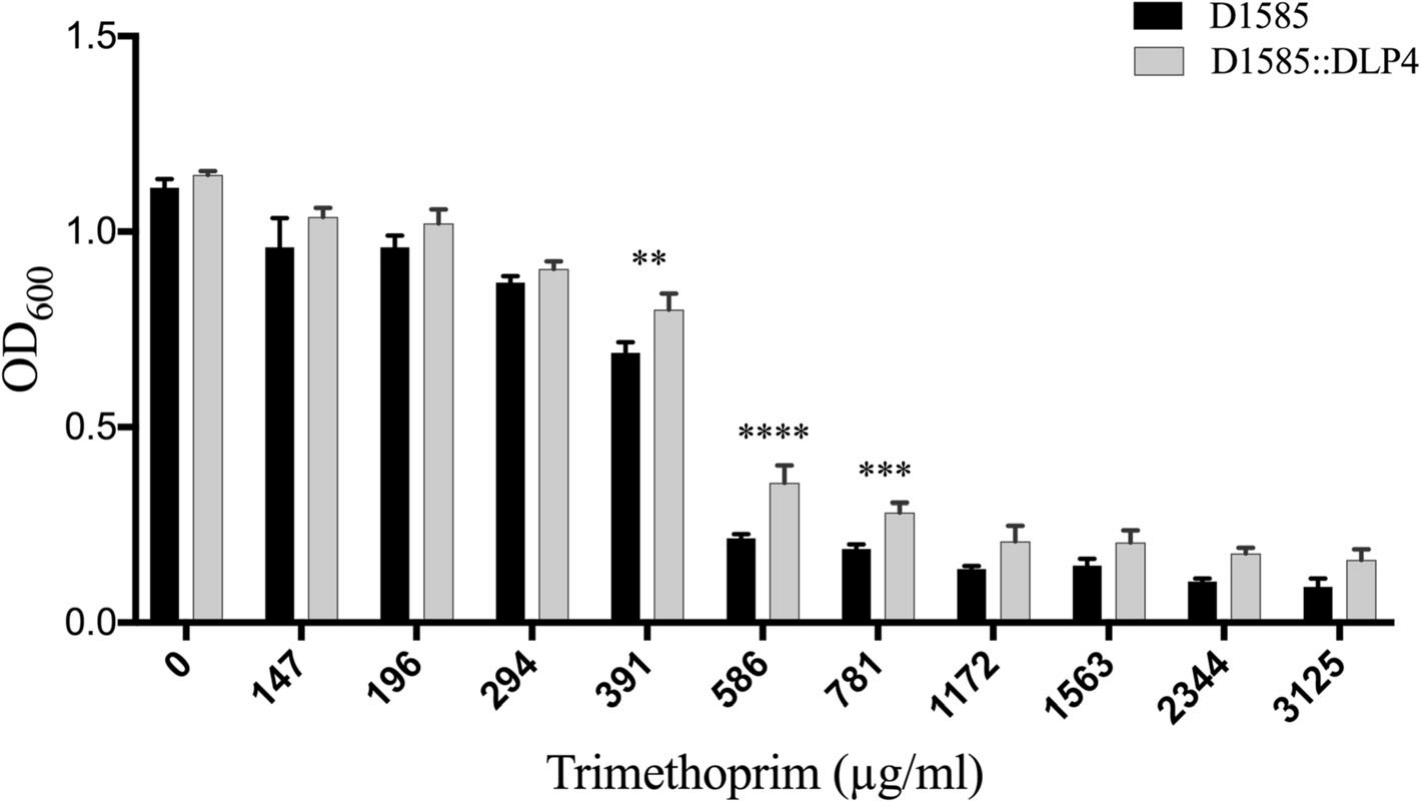
Trimethoprim resistance of strain D1585::DLP4 lysogen increases as compared to the strain D1585 wildtype control. Assay was completed in biological and mechanical triplicate. Two-way ANOVA with Sidak’s multiple comparisons test was performed on the MIC data, and statistical significance is represented as: ****, *P* < 0.0001; ***, *P* < 0.001, ** *P* < 0.01

**Fig. 5:**
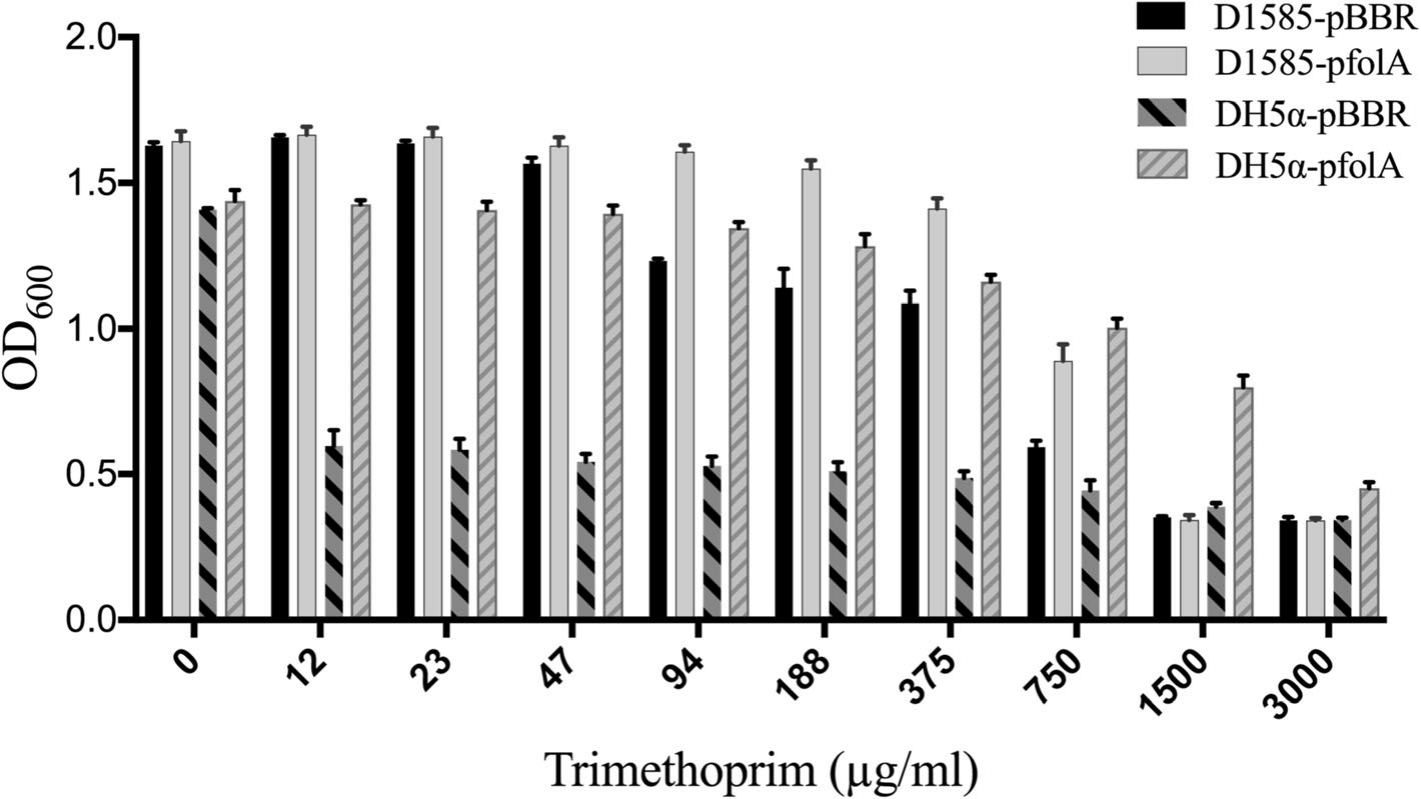
Increase of trimethoprim resistance in *E. coli* DH5휶 containing DLP4 *folA*. Trimethoprim resistance in *E. coli* DH5α increases from < 12 μg/ml to an LD_90_ of 3000 μg/ml when DLP4 *folA* is expressed from the pBBR1MCS plasmid as compared to an empty vector control

**Fig. 6:**
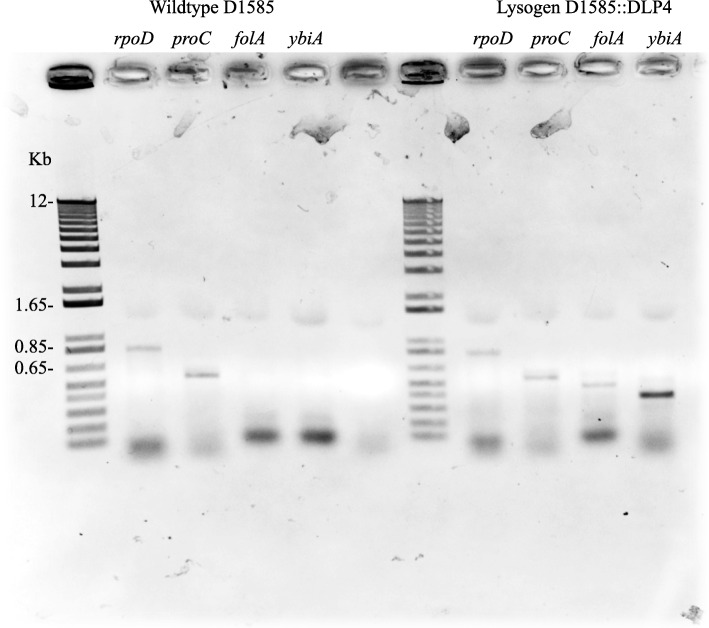
Reverse transcription PCR detects expression of *folA* and *ybiA* genes in the D1585::DLP4 lysogen compared to wildtype D1585 control. Positive controls are the exponential sigma factor-encoding gene *rpoD* and Pyrroline-5-carboxylate reductase-encoding gene *proC*. A 1% agarose gel was used to run the RT-PCR reaction and an Invitrogen 1 kb plus DNA ladder was used for size comparison

The discovery that DLP4 encodes *folA* (BIT20_024) was surprising at first, but in the context of the surrounding genes involved in deoxyribonucleoside generation [50], its genomic location is fitting. I-TASSER analysis of DLP4 FolA predicted a structural similarity to *Bacillus anthracis* DHFR (FolA: TM-score 0.919, coverage 0.963). As FolA is responsible for trimethoprim resistance in bacteria, it was important to investigate whether the DLP4 encoded *folA* produced a functional FolA causing lysogenic conversion of the host bacterium. Comparing the resistance profile of strains D1585 to D1585::DLP4, there is a statistically significant increase in trimethoprim resistance at 391 (*P* value 0.0003), 586 (*P* value < 0.0001) and 781 (*P* value 0.004) μg/ml concentrations (Fig. 4). It is apparent from Fig. 4 that the presence of FolA from DLP4 contributes to the overall trimethoprim resistance both below (391μg/ml) and above (586 μg/ml) the antibiotic breakpoint. Although the host strain D1585 is resistant to trimethoprim, there is a small but significant increase in trimethoprim resistance provided by the prophage FolA gene expression. To confirm that the DLP4 FolA is functional, the DLP4 *folA* gene was cloned into the pBBR1MCS plasmid and expressed in *E. coli* DH5α and *S. maltophilia* D1585 against an empty vector control in varying concentrations of trimethoprim (Fig. 5). The results confirm that the DLP4 FolA is functional, causing an increase in resistance to trimethoprim when expressed. The DLP4 FolA protein increased the trimethoprim LD90 for DH5α-p*folA* strain to 3000 μg/ml from the < 12 μg/ml observed with the empty vector control. To confirm increased trimethoprim resistance observed for the DLP4 lysogen was specifically due to expression of *folA* in the lysogen, reverse transcription PCR (RT-PCR) analysis was performed. Positive controls for the RT-PCR utilized gene-specific primers designed against D1585 housekeeping genes *rpoD* (σ70) and *proC* (proline biosynthetic gene) [51]. The RT-PCR results show that *folA* is expressed during lysogeny (Fig. 6) and explains the observation of increased trimethoprim resistance of the lysogen as compared to the wildtype control.

### Virion morphogenesis module

The virion morphogenesis module of DLP4 encodes 27 ORFs, and BLASTP results against these proteins provided close matches for all but one protein, BIT20_072 (Fig. 3, Table 3). Of the 27 predicted ORFs within the module, 11 have high homology to the *Pseudomonas* phage PaMx74. These encoded proteins include three hypotheticals, a tape measure protein (BIT20_081), a putative tail terminator protein (BIT20_077), a putative FAD/FMD-containing dehydrogenase (BIT20_003), four putative virion structural proteins (BIT20_004, BIT20_006, BIT20_074, and BIT20_075) and a serine-glyoxylate aminotransferase (BIT20_002). The phage AAT-1 BLASTP-aligned homologs include five of the 27 DLP4 proteins: two hypothetical proteins, the portal protein (BIT20_061), a putative structural protein (BIT20_064) and the major tail structural protein (BIT20_078). The tail assembly gene products of DLP4 (BIT20_007–009) are most similar to two *Xylella* phages named Salvo and Sano [52]. The ORF following the tail assembly genes of DLP4 encodes a tail fiber protein (BIT20_010), which is most homologous to *Xylella* phage Salvo, though a CD-Search does not identify a tail fiber domain in this protein. There are four phage PaMx28 BLASTP-aligned homologs in the DLP4 genome: the major and minor head proteins (BIT20_071 and BIT20_062 respectively), a hypothetical protein (BIT20_063) and one virion structural protein (BIT20_076). When looking at the DLP4 virion morphogenesis module, it appears that there has been an abundance of recombination between phages such as PaMx74, PaMx28, Salvo, Sano and AAT-1 that has resulted in DLP4 encoding many distinct regions of homology at the protein level to each of these five phages.

### Lysis module

The lysis module (Fig. 3, Table 3) of this bacterium directly follows the virion morphogenesis module and is composed of five ORFs (BIT20_011–015). The first gene in the lysis module encodes the holin protein (BIT20_011) that is predicted to be a class II holin due to the presence of two transmembrane domains. The next gene encodes a predicted endolysin (BIT20_012) with a conserved D-alanyl-D-alanine carboxypeptidase identified by CD-Search. This domain is also featured in DacA of *E. coli* K-12 and is responsible for trimming the carboxy-terminal D-alanyl moieties from the peptidoglycan pentapeptides [53]. The next two ORFs (BIT20_013/014) following encode i- and o- spanins respectively. The two spanins associate together in the periplasm and physically link the inner membrane to the outer membrane. The i-spanin is tethered to the inner membrane near the N-terminal domain through a transmembrane region and the C-terminal domain associates with the o-spanin in the periplasm [54]. The o-spanin contains a modified N-terminal Cys residue, which has added fatty acid and diacylglycerol groups to anchor the lipoprotein to the outer membrane, allowing the C-terminal domain to associate with the C-terminal domain of the i-spanin [54]. The final ORF of the lysis cassette (BIT20_013) is predicted to contain a transmembrane domain and a conserved domain belonging to the SpsE protein superfamily, more specifically to the NeuB_NnaB (TIGR03569) family. The NeuB_NnaB family consists of functional N-acetylneuraminate synthase proteins, which produce N-acetylneuraminic acid (NANA), a sialic acid that is used by bacterial pathogens to hide from their mammalian hosts [55]. It is functionally unclear why this gene is present within the lysis module of the DLP4 genome, however this pattern is observed within lysis modules of other phages. Three *Pseudomonas* phages (PaMx74, AAT-1, PaMx28) and one *Xanthomonas* phage (Xoo-sp246) encode this same set of genes [56].

### YbiA operon

Within the virion morphogenesis region, there is an insert of approximately 2750 bp in the reverse frame encoding six genes (BIT20_065–070) (Fig. 3, Table 3). This insert also exhibits a reduction in GC content from the surrounding 66 to 61%. The operon is under the control of a single promoter located 65 bp upstream of the first gene of the insert, BIT20_070. The six genes encoded in this operon are also syntenic with three other phages (*Xanthomonas* phage Xoo-sp2 and *Pseudomonas* phages PaMx28 and AAT-1) in the same orientation. The *Pseudomonas* phage PaMx74 contains a single gene (PaMx74_15) that exhibits homology with BIT20_066 found within the DLP4 operon, though the surrounding genes of PaMx74_15 do not have homology to the rest of the genes within the DLP4 operon.

The BIT20_070 gene product does not exhibit homology with any BLASTP entries, and I-TASSER analysis did not provide significant hits with high confidence. The gene product of BIT20_069 showed homology with a hypothetical protein AAT1_02013 from *Pseudomonas* phage AAT-1, but no conserved domains were identified for this protein using CD-Search. BIT20_068 does not exhibit homology with any BLASTP entries when limited to viruses, but with no database restrictions, BIT20_068 exhibited some homology to a hypothetical multiple-species protein within *Enterobacteriaceae*. BLASTP analysis of BIT20_67 gene product showed homology to hypothetical protein AAT1_02012 belonging to *Pseudomonas* phage AAT-1 (73% coverage, 2.0E-19, 49% coverage). I-TASSER structural prediction of the protein identified similar possible structural domains. The top homolog provided by I-TASSER (TM-score 0.705 and coverage 0.952) was an anti-sigma factor antagonist with the protein data bank ID of 3ZTA. This *Moorella thermoacetica* protein has been shown to be involved in the bacterial stressosome, which is responsible for controlling secondary messenger signaling [57]. For BIT20_066, high homology was exhibited to the hypothetical protein PaMx74_15 from *Pseudomonas* phage PaMx74. I-TASSER analysis of this protein predicts that it is structurally similar to the protein data bank entry 1FOH (TM-score 0.875 and coverage 0.937), a phenol hydrolase from *Trichosporon cutaneum* within the Fungi Kingdom.

**Fig. 7:**
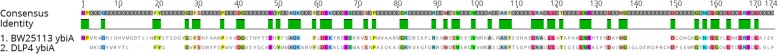
Protein alignment of DLP4 YbiA against *Escherichia coli* BW25113 YbiA. I-TASSER structure assembly simulation data generated on DLP4 YbiA was submitted to TM-align, which returned a close predicted structural homology to *E. coli* protein YbiA (TM-score 0.896 and coverage 0.913). Enzymatically important amino acids are located at positions 48 (E), 92 (W), 133 (D) and 136 (W)

The final gene within the operon (BIT20_065) is predicted to encode a putative YbiA-like protein from *Pseudomonas* phage PaMx28. YbiA is responsible for the swarming phenotype within *E. coli* K-1249 [57]. I-TASSER analysis of the DLP4 YbiA-like protein produced a TM-score of 0.896 and coverage of 0.913 to the YbiA protein of *E. coli*. A MUSCLE protein alignment between the two proteins shows a breakdown in sequence homology at the protein level. Although known functional domains of *E. coli* YbiA (amino acids Glu48; Trp89; Asp130; and Trp133) are still present in the DLP4 protein sequence, the numbering for the last three amino acids changes to 92, 133 and 136 within the alignment (Fig. 7, [58]). Analysis of the annotation results overall suggests that this operon encodes moron genes which may help the host cell respond to environmental stress. This is the sixth of 15 *S. maltophilia* phage characterized to carry moron genes [10, 13, 15–17], which can potentially lead to the lysogenic conversion of *S. maltophilia* strains, suggesting that prophage expression of genes is widespread in the SMC [59].

### Investigation of swarming phenotype

**Fig. 8:**
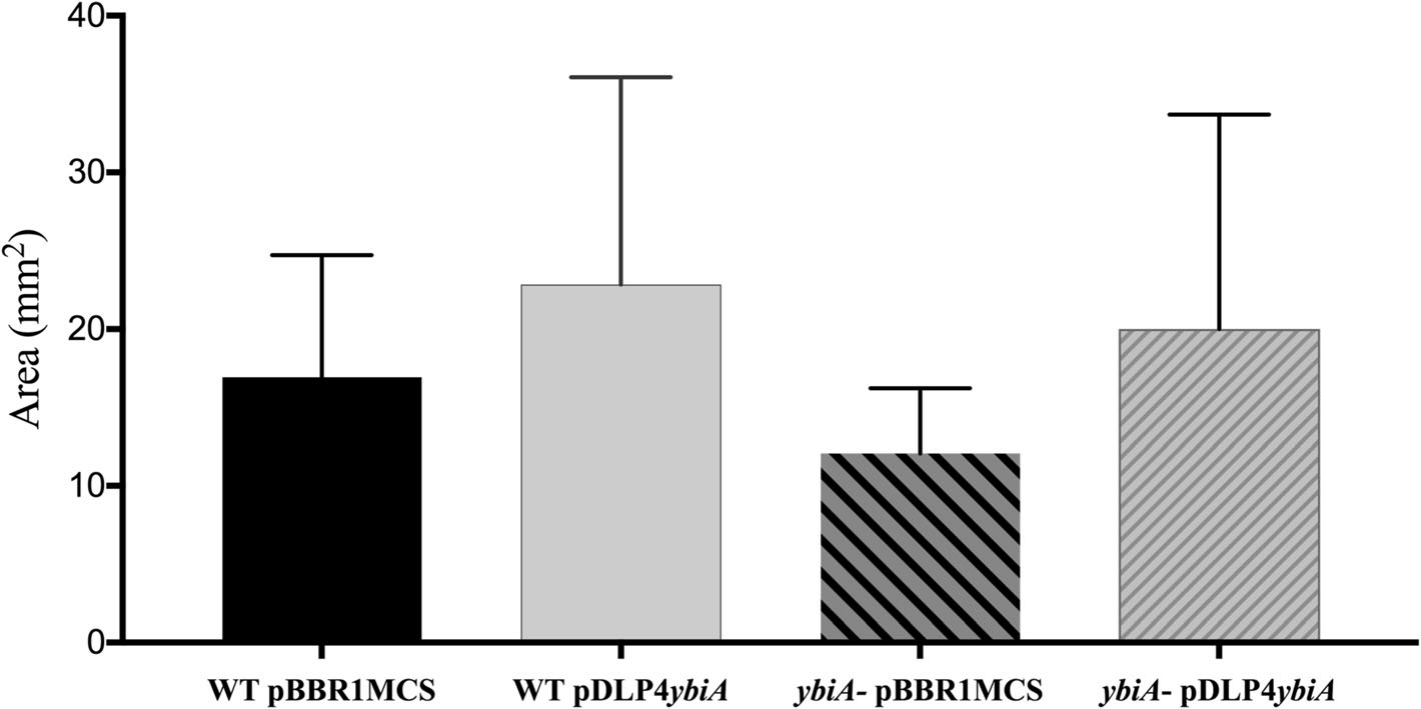
Complementation of *E. coli* BW25113 *ybiA*^−^ with DLP4 *ybiA* restores swarming phenotype. Image data from three biological and mechanical triplicate swarming experiments was used to measure swarm area using ImageJ. Plasmids used in this experiment were pBBR1MCS and pBBR1MCS with DLP4 *ybiA* inserted (pYbiA). Area averages and standard deviation were calculated and graphed in GraphPad Prism

Identification of the *ybiA* gene within a small operon of phage DLP4 raised questions about the ability of the YbiA-like protein to affect swarming within strain D1585. The swarming ability of *S. maltophilia* is currently under investigation, with conflicting reports presented in recent literature [60–62]. One study showed *S. maltophilia* is capable of what appeared to be swarming, though it was instead found to be flagella-independent translocation in the presence of extracellular fatty acids [60]. Observation of swarming on plates inoculated with wild-type D1585, the lysogen and D1585 containing cloned DLP4 pYbiA and empty vector control (pBBR1MCS) did not show any swarming phenotype (data not shown). However, the predicted structural similarity of DLP4 YbiA protein to *E. coli* YbiA suggests that DLP4 *ybiA* might potentially complement a *ybiA*- *E. coli* mutant; therefore, swarming experiments were conducted in *E. coli* strains BW25113 and *ybiA*770(del)::kan. These swarming results indicate that the phage DLP4 encoded YbiA can complement the *E. coli ybiA*- knockout to wildtype swarming levels (Fig. 8). Repeated swarming assays did not reduce the considerable variation observed within the biological and mechanical replicates, though it is important to note that the variation in swarming was observed in all strains studied. RT-PCR of wildtype *S. maltophilia* D1585 and the D1585::DLP4 lysogen showed *ybiA* is expressed in the lysogen (Fig. 6), but its expression did not enhance the swarming phenotype of D1585::DLP4 as it did with the *E. coli* strains (data not shown).

## Conclusions

Genomic characterization of the novel temperate *S. maltophilia* phage DLP4 reveals a restriction enzyme resistant genome 63,945 bp in size encoding 82 potential ORFs. The GC % content of the DLP4 genome is found to be reflective of the host GC content of 65%. Phage DLP4 encodes a near complete deoxynucleoside conversion and salvage pathway including a functional dihydrofolate reductase which was shown to be functional and expressed in the lysogen. The DLP4 encoded YbiA operon has a functional YbiA protein that is required for the swarming phenotype of *E. coli* and is expressed during the lysogenic cycle, though no swarming was observed for *S. maltophilia* strain D1585. This operon also encodes proteins that may be involved in a bacterial stress response, such as a putative phenol hydrolase and an anti-sigma factor antagonist homolog involved in the bacterial stressosome. A putative Cas4 nuclease is encoded within the DNA replication and repair module of DLP4, though the role of this protein in the DLP4 infection cycle is unknown, and it is not expressed during the lysogenic cycle. Although phage DLP4 is likely not suitable for therapeutic use due to its temperate lifecycle and the presence of moron genes, molecular modification of the DLP4 genome could potentially optimize this phage for therapeutic applications. As this is the sixth of 15 characterized *S. maltophilia* phages discovered to carry moron genes, it is anticipated that lysogenic conversion in *S. maltophilia* is common. Further research into temperate *S. maltophilia* bacteriophages will help elucidate the role that these phages play in the virulence and antibiotic resistance of *S. maltophilia* isolates.

## Abbreviations

% GC: Percent guanine: cytosine
cDNA: Complementary DNA
CRISPR: Clustered Regularly Interspaced Short Palindromic Repeats
DHFR: Dihydrofolate reductase
DNA: Deoxyribonucleic acid
EtOh: Ethanol
LB: Lysogeny broth
MH: Mueller Hinton
RNA: Ribonucleic acid
RT-PCR: Reverse transcription polymerase chain reaction
SM: Suspension media
SMC: *Stenotrophomonas maltophilia* complex
